# Enoxaparin attenuates doxorubicin induced cardiotoxicity in rats via interfering with oxidative stress, inflammation and apoptosis

**DOI:** 10.1186/s40360-017-0184-z

**Published:** 2018-01-10

**Authors:** Reem Ali Shaker, Samer Hassan Abboud, Hayder Chasib Assad, Najah Hadi

**Affiliations:** 1 Najaf Health Directorate, Ministry of Health, Najaf Governorate, Iraq; 2 Faculty of Medicine, University of Kufa, Najaf Governorate, Iraq; 3 Department of Clinical Pharmacy and Therapeutics, Faculty of Pharmacy, University of Kufa, Najaf Governorate, Iraq

**Keywords:** Doxorubicin, Cardiotoxicity, Low molecular weight heparin, Antioxidant, Inflammatory markers and caspase-3

## Abstract

**Background:**

Doxorubicin (DOX) is commonly used in the treatment of many types of cancers but its cardiotoxicity is limiting its clinical use. Beyond its anticoagulant action, enoxaparin (ENX), a low molecular weight heparin, has been shown to exert multiple pharmacological actions including antioxidant, anti-inflammatory and antiapoptotic effects. Therefore, the current study aimed to assess if ENX could ameliorate cardiotoxicity induced by DOX.

**Methods:**

Twenty-one adult male Wistar albino rats were randomly allocated into three groups (*n* = 7 each) of control, receiving 0.9% saline (i.p.), DOX, receiving 2.5 mg/kg of DOX (i.p.) thrice weekly; and DOX + ENX, receiving ENX (250 IU/kg/day i.p.) and a DOX dose equivalent to that of the DOX only group.

**Results:**

DOX-induced cardiotoxicity was indicated by marked increases in cardiac troponin I (cTnI) and severe histological lesions, which significantly correlated with cardiotoxicity, oxidative stress, inflammation and apoptosis markers, compared to controls. DOX group also showed elevations in malondialdehyde (MDA), a marker of oxidative stress, and reductions in total antioxidant capacity (TAC). Cardiac inflammatory markers including tumor necrosis factor alpha (TNF-α) and interleukin-1 beta (IL-1β) and caspase-3, an apoptotic marker, were also elevated in the DOX group. DOX, however, did not significantly alter brain natriuretic peptide (BNP) levels. ENX significantly attenuated, but not completely reversed, DOX-induced cardiotoxicity through lowering cTnI and improving cardiomyopathy histopathological scores as compared to the DOX group. ENX also decreased MDA, increased TAC of rats’ heart to levels relatively comparable to control. Significant reductions in TNF-α, IL-1β and caspase-3 were also observed following ENX treatment relative to the DOX only group.

**Conclusions:**

Collectively, these results describe a cardioprotective effect for ENX against DOX-induced cardiotoxicity which is likely facilitated via suppression of oxidative stress, inflammation and apoptosis.

## Background

Doxorubicin (DOX) is an antineoplastic drug that belongs to anthracyclines and considered among the most effective oncology agents ever developed [1, 2]. DOX has been the mainstay of cancer chemotherapy for decades, with well-established, highly effective and notable successes in the treatment of both solid and hematological malignancies for adult and pediatric patients [3]. The clinical effectiveness of DOX, however, is hindered by its most significant and dose-dependent cardiotoxicity [4, 5], with recent reports showing that DOX-induced cardiomyopathy being encountered more frequently due to growing survival rates in cancer patients treated by DOX-based chemotherapy [6].

Multifactorial pathways are implicated in the mechanisms of cardiotoxicity induced by DOX [7, 8]. There is a large body of evidence to show that oxidative stress, inflammation and apoptosis are possibly involved [4]. Increased oxidative stress and continuous release of reactive oxygen species (ROS) via many contributing pathways are key elements to DOX-induced cardiotoxicity. These ROS can stimulate lipid peroxidation and subsequent oxidative damage to mitochondria and cell membranes of myocytes [9]. Increased oxidative stress after DOX exposure also leads to expression of transcription factor, nuclear factor kappa B cell (NF-κB) and activation of nucleotide-binding oligomerization domain (NOD)-like receptor protein 3 (NLRP3) inflammasome, which, consequently, increases release of proinflammtory cytokines in the myocardium such as tumor necrosis factor alpha (TNF-α) and interleukin-1 beta (IL-1β) [10, 11]. There is also evidence which indicates that both extrinsic and intrinsic pathways of apoptosis are involved in the pathogenesis of DOX-Induced cardiotoxicity. Doxorubicin triggers apoptosis either directly by localizing to the mitochondria [12] and/or through oxidative stress and high accumulation of calcium in the cell, both are known to enhance the release of cytochrome C and stimulate the final step in caspase-3 induced programmed cell death [4, 8, 13].

In clinical settings, cardiotoxicity of DOX presents an enormous challenge in the face of its proven chemotherapeutic effectiveness, which therefore calls for establishing more rigorous approaches to prevent or limit DOX-mediated side effects. Recently, it has been shown that cardiotoxic effects of DOX were ameliorated by regular unfractionated heparin treatment and that effects were mediated through reductions in NF-κB protein expression and inflammatory mediators [14], suggesting additional therapeutic benefits for heparins beyond their anticoagulant action. Indeed, heparins have been shown to retain anti-antioxidant and free radicals scavenging effects [15, 16], anti-inflammatory and immunomodulatory properties [17, 18], and cytoprotective and anti-apoptotic activities [19, 20]. Among heparins, the low molecular weight heparin (LMWH) enoxaparin (ENX) has been frequently and routinely used in clinical practice perhaps due to its less side effect profile compared with unfractionated heparin, cost effectiveness, broadest range of FDA-approved indications, preferential effects on factor Xa and greater bioavailability relative other LMWHs [21]. Like regular heparin, ENX has been shown, in rats [22], to exert powerful antioxidant effects, which is reported to be due to its ability to prevent lipid peroxidation. Furthermore, ENX has demonstrated a clear anti-inflammatory benefit and is considered the most active in this regard among LMWHs [23, 24]. However, yet to be explored is if ENX would be able to prevent, reverse or at least ameliorate the deleterious cardiac effects of DOX and therefore improve tolerability of this widely used chemotherapeutic agent. Accordingly, the present study was designed to investigate the cardioprotective effects of ENX against DOX-induced cardiotoxicity in rats and identify some possible underlying pathways that might be involved.

## Methods

### Animals

A total of 21 adult male Wistar albino rats weighing 150–250 g were bred in and obtained from the Faculty of Science, University of Kufa. The animals were placed in the animal house facility of the Faculty of Pharmacy of the University of Kufa. The animals were housed in an air-conditioned room, with temperatures maintained at 25 ± 2 C° and a 12-h dark/light cycle. The animals were supplied with water and standard rat chow ad libitum. Rats were acclimatized for 2 weeks in the animal house prior to their use in the experiments. The study was conducted according to the national guidelines for the Care and Use of Laboratory Animals. All protocols were approved by the High Committee for Review and Approval of Research Proposals of the Faculty of Medicine of the University of Kufa (Ref#2015–19).

### Study design

Twenty one adult male Wistar albino rats were randomly divided into three groups:

1. Control group (*n* = 7), which received 0.5 ml of 0.9% saline intraperitoneally (i.p.) daily over two weeks.
2. DOX group (n = 7), which received doxorubicin HCL (Adriamycin, Pfizer Australia) at a dose of 2.5 mg/kg (i.p.) thrice weekly for two weeks (cumulative dose 15 mg/kg).
3. DOX plus ENX group (n = 7), in which DOX was injected in the same manner as described in group 2 but rats were also administered with ENX (Clexane, Sanofi aventis, France) i.p. in a dose of 250 IU/kg/daily over two weeks as described previously [25, 26].

During the treatment period, one rat died from DOX and DOX plus ENX groups, and therefore data from these groups were collected from 6 animals per group. No deaths were observed within the control group.

### Serum and tissue preparation

Body weight for each animal was measured before the treatment and after 48 h from the last DOX dose. Animals were then anesthetized with a mixture of 100 mg/kg of ketamine (Duopharma, Malaysia) and 10 mg/kg of xylazine (Alfasan woerden, Holland) (i.p.). Blood samples were then collected directly from the left ventricle of the heart by heart puncture and serum was obtained by centrifugation at 3000 rpm for 10 min. Following blood collection, animals were sacrificed by a thoraco-abdominal incision and the heart was removed, washed by ice-cold saline then weighed. The heart tissues were cut in transvers section into two parts. The basal parts were snap-frozen using dry ice until later use for tissue homogenization, which was carried out in 0.1 M phosphate buffered saline (pH 7.4) containing 1% Triton-100 and protease inhibitor cocktail and processed using high intensity ultrasonic liquid processor. The homogenates were centrifuged at 4 C^o^ and supernatants were used for determination of tissue markers. The apical parts of the tissue sections were fixed in 10% neutral buffered formalin for 48 h. The specimens were then dehydrated by passing through graded concentrations of alcohol, cleared by xylene to remove alcohol, embedded in paraffin and then allowed to harden. The paraffin blocks were subsequently cut into 5-μM thickness sections with a microtome and sections were transferred into a water bath where they were allowed to float. Floating sections were then mounted onto microscope slides, dried in an oven at 60 C^o^ for 20 min, stained by hematoxylin-eosin (H&E) and examined for histopathological changes using light microscopy (Olympus CX31).

### Enzyme immunoassay

The cardiotoxicity indices including brain natriuretic peptide (BNP) and cardiac troponin I (cTnI) were determined in serum using a rat ELISA kit (Cloud-clone corp., USA). The markers of oxidative stress, malondialdehyde (MDA) and total antioxidant capacity (TAC), were determined in cardiac tissue homogenates using MDA ELISA kit (Cloud-clone corp. USA), and OxiSelect™ TAC assay kit (Cell Biolabs, USA), respectively. The antioxidant capacity is determined relative to uric acid standards and therefore results were expressed as mM uric acid. Cardiac tissue homogenate was further used to assess inflammatory markers (TNF-α and IL-1β) and the apoptotic marker caspase-3 using their respective ELISA kits (Cloud-clone corp., USA).

### Histopathological examination

The histology slides were examined by light microscopy (Olympus CX31) to assess histopathological changes and their severity according to the method of Saad et al. [27]. Tissue pathological changes within each slide were scored as follows: (A) Interstitial edema and myocardial fiber swelling (1+), (B) myocardial fiber disorganization with or without fibroblastic proliferation (1+), (C) myocardial fiber vacuolization or perinuclear vacuolization) (1+), (D) myocytolysis/necrosis of myocardial fibers (1+), and when there was no damage noted (0). The cardiomyopathy (CMY) severity scores were graded such that 0 represented no damage, 1 represented mild lesion, 2 represented moderate lesion and 3 or more represented severe lesion.

### Data and statistical analyses

Statistical analyses were performed using GraphPad Prism software (GraphPad Prism software v6 Inc., La Jolla, CA, USA). All data is expressed as mean ± standard error mean (SEM). A one-way Analysis of Variance (ANOVA) was used to compare all three groups followed by Fisher’s least significant difference (LSD) post-hoc test. Baseline and terminal body weight within each group and between groups was compared using a two-way ANOVA followed by Fisher’s least significant difference (LSD) post-hoc test. Data from all three groups was also pooled together and the cardiotoxicity, oxidative stress, inflammation and apoptosis markers were correlated against cardiomyopathy (CMY) severity scores using a Pearson correlation analysis followed by linear regression. The relative heart weight index was calculated as heart weight-to-body weight ratio. In all tests, *P* ≤ 0.05 was considered to be statistically significant.

## Results

### Effect of ENX on body weight, heart weight and the ratio of the heart to body weight

As shown in Table 1, body weight did not significantly increase within the control group (*P* = 0.517). DOX and DOX+ ENX groups showed minor drops in body weight but this did not approach statistical significance (*P* > 0.05). As a result, baseline and terminal body weights did not markedly differ between groups. Heart weight was significantly lower in the DOX group compared to controls (*P* = 0.007). Heart weight in the ENX-treated group tended to show a similar trend but this did not approach statistical significance (*P* = 0.064). When heart weights were corrected to body weight ratios did not significantly differ between groups.

**Table 1:** Effect of ENX on baseline body weight, heart weight and the ratio of the heart to body weight

Group	Baseline BW(g)	Terminal BW (g)	Heart weight (g)	Heart/BW
Control ^n = 7^	196 ± 10	205 ± 8	0.83 ± 0.054	4.1 ± 0.23
DOX ^n = 6^	202 ± 14	177 ± 12	0.65 ± 0.278^a^	3.7 ± 0.21
ENX plus DOX ^n = 6^	216 ± 10	194 ± 5	0.71 ± 0.25	3.6 ± 0.10

*BW* body weight. Data is expressed as mean ± SEM. ^a^ *P* ≤ 0.05 for DOX vs. control (analyzed by one-way ANOVA followed by Fisher’s least significant difference (LSD) test)

### Effect of ENX on cardiotoxicity indices (cTnI and BNP)

**Fig. 1:**
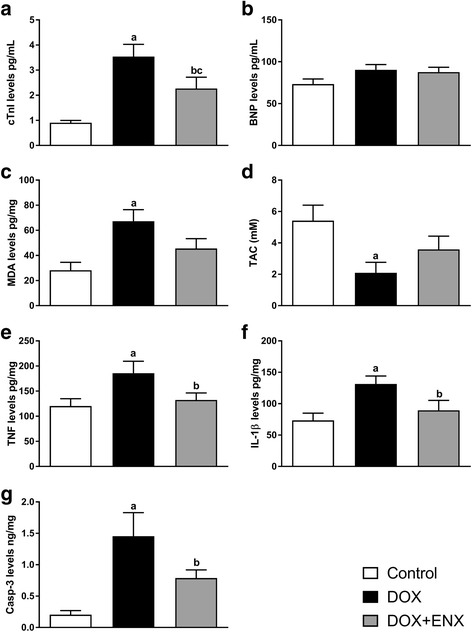
Effects of enoxaparin (ENX) treatment on cardiotoxicity, antioxidant, inflammatory and apoptosis parameters on doxorubicin (DOX)-induced cardiomyopathy in male Wistar albino rats. Graph illustrates changes in (**a**) serum levels of cardiac troponin I (cTnI), (**b**) serum levels of brain natriuretic peptide (BNP), (**c**) cardiac tissue malondialdehyde (MDA), (**d**) cardiac tissue total antioxidant capacity (TAC), (**e**) cardiac tissue tumor necrosis factor alpha (TNF-α), (**f**) cardiac tissue interleukin 1-beta (IL-1β) and (**g**) cardiac tissue caspase-3 (casp-3) in control (*n* = 7), DOX (*n* = 6) and DOX plus ENX (*n* = 6) experimental groups. All data is presented as mean ± SEM, analyzed by one-way ANOVA followed by Fisher’s least significant difference (LSD) test. ^a^ *P* ≤ 0.05 for DOX vs. control; ^b^ *P* ≤ 0.05 for ENX plus DOX vs. DOX and ^c^ *P* ≤ 0.05 for ENX plus DOX vs. control

Administration of DOX caused a significant increase (*P* < 0.001) in the serum level of cTnI as compared to levels measured within the control group. Treatment with ENX produced pronounced reductions (*P* = 0.017) in serum concentration of cTnI relative to the DOX group; however, ENX administration did not completely revert cTnI concentrations back to control levels (*P* = 0.029) (Fig 1a). BNP level, in contrast, was not significantly different among all groups (Fig. 1b).

### Effect of ENX on oxidative stress markers (MDA and TAC) in cardiac tissue

DOX administration produced a marked elevation (*P* = 0.002) in MDA levels (Fig 1c), which was also associated with a significant (*P* = 0.015) reduction in cardiac TAC (Fig 1d) relative to controls. Treatment with ENX tended to reduce cardiac MDA but levels did not approach statistical significance (*P* = 0.069). Importantly, however, MDA concertation in the ENX-treated group was reduced down to levels not significantly different (*P* = 0.127) to controls (Fig 1c). In contrast, TAC measured within cardiac tissue homogenate of ENX + DOX group was upregulated to levels comparatively similar (*P* = 0.150) to those of the control group, but with measures remaining relatively comparable (*P* = 0.255) to those observed in the DOX group (Fig 1d).

### Effect of ENX on inflammatory markers (TNF-α and IL-1β) in cardiac tissue

Cardiac TNF-α and IL-1β were significantly (both *P* < 0.05) higher in the DOX group relative to controls (Fig. 1e-f, respectively). ENX treatment lowered both TNF-α and IL-1β concentrations compared with the DOX group (both *P* ≤ 0.05) and, most importantly, levels paralleled (both *P* > 0.05) those measured within control rats (Fig. 1e-f, respectively).

### Effect of ENX on the apoptotic marker caspase-3 in cardiac tissue

DOX administration resulted in a significant increase (*P* < 0.001) in cardiac caspase-3 levels relative to the control group (Fig. 1g). ENX administration decreased (*P* = 0.05) tissue caspase-3 as compared to the DOX group, and levels compared relatively closely (*P* = 0.078) with those measured in control animals (Fig. 1g).

### Effect of ENX on myocardial histopathology

**Fig. 2:**
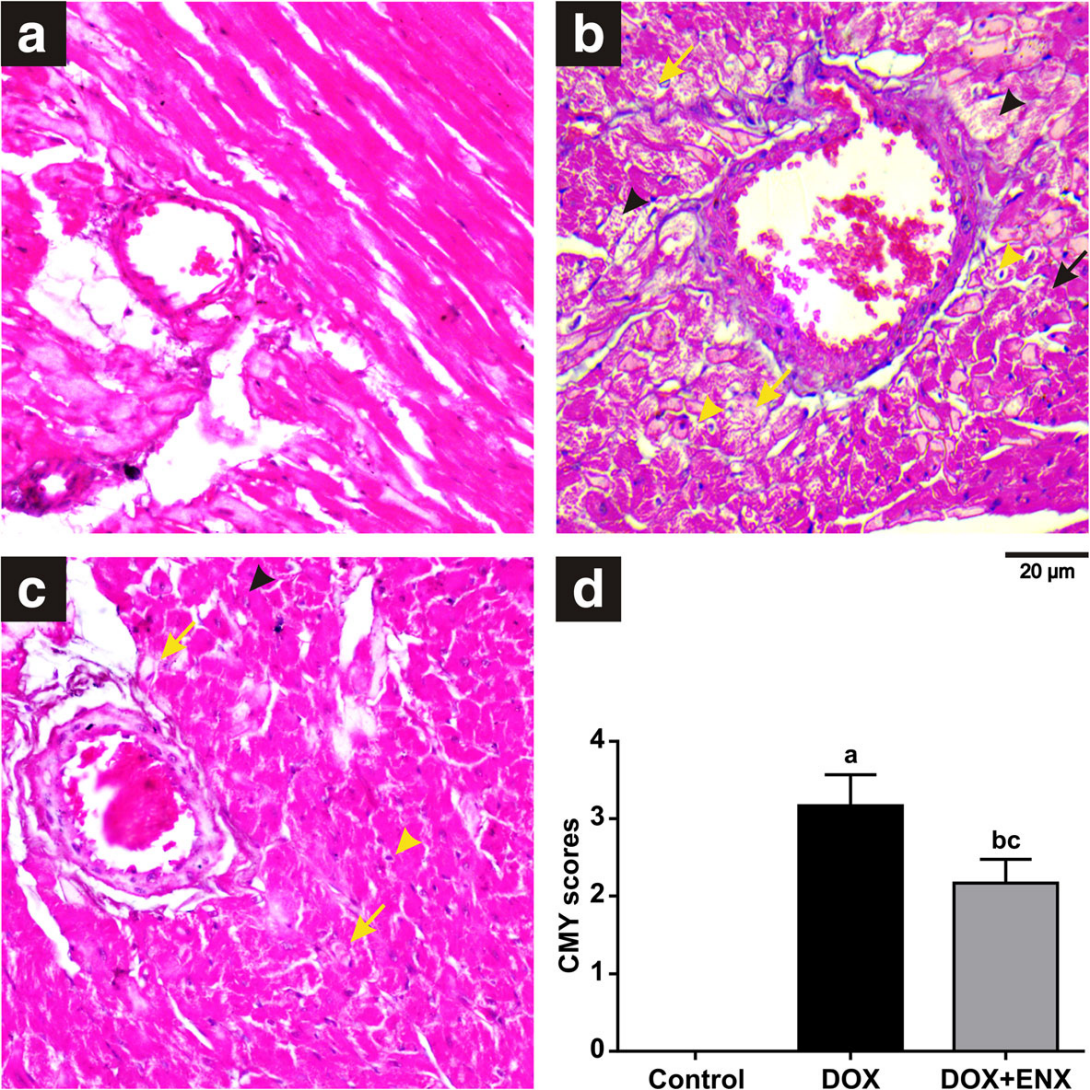
Photomicrographs of myocardium sections taken at 400X magnification (**a**–**c**) and cardiomyopathy (CMY) severity scores graded from 0 to 4: 0 represents no lesion, 1 represents mild lesion, 2 represents moderate lesion and 3 or more represents severe lesion. **a** Control group which shows normal architecture of myocardium (score range = 0), (**b**) Doxorubicin (DOX) group which demonstrates severe lesion with congestion of myocardial vessels, disorganization of myocardial fibers, myocardial fiber swelling (black arrow), myofibrillar loss (black arrowhead) and vacuolization of the cytoplasm (yellow arrow) and perinuclear vacuolization (yellow arrowhead) (score range = 3–4), and (**c**) Enoxaparin (ENX) plus DOX group which shows mild to moderate lesion that is characterized by disorganization of myocardial fibers and myocardial fiber swelling (black arrow) with less cytoplasm (yellow arrow) and perinuclear vacuolization (yellow arrowhead) and no myofibrillar loss (score range = 1–3), and (D) Overall CMY scores presented as mean ± SEM., analyzed by one-way ANOVA followed by Fisher’s least significant difference (LSD) test. ^a^ *P* ≤ 0.05 for DOX vs. control; ^b^ *P* ≤ 0.05 for ENX plus DOX vs. DOX and ^c^ *P* ≤ 0.05 for ENX plus DOX vs. control

The histology of heart tissue in the control group showed normal morphological appearance (Fig. 2a). Rats in the DOX group, however, showed severe pathological lesions, which were characterized by congestion of myocardial vessels, myocardial swelling, myocardial fibers disorganization, cytoplasmic vacuolization, perinuclear vacuolization, and myofibrillar loss (Fig. 2b). The lesions of ENX group, in contrast, scored as mild to moderate and displayed less cytoplasmic or perinuclear vacuolization and no myofibrillar loss Fig. 2c. As shown in Fig. 2d, overall CMY scores in the DOX group were significantly higher compared to controls (*P* < 0.001). In the ENX-treated group, myocardial lesions were markedly attenuated (*P* = 0.024) as compared to the DOX group but were not completely prevented when compared to controls (*P* < 0.001). Most importantly, CMY scores correlated positively with cTnI, BNP, MDA, TNF-α, IL-1β and caspase-3 while a significant negative correlation between CMY scores and TAC was seen (Table 2).

**Table 2:** Correlation between cardiotoxicity, oxidative stress, inflammation and apoptosis markers against cardiomyopathy (CMY) severity scores in male Wistar albino rats (*n* = 19)

Parameter	cTnI	BNP	MDA	TAC	TNF-α	IL-1β	Casp-3
Pearson r	0.627	0.591	0.635	−0.575	0.690	0.579	0.748
R square	0.393	0.349	0.404	0.330	0.477	0.335	0.560
*P* value	0.0040	0.008	0.004	0.010	0.001	0.009	0.0002

*cTnI* cardiac troponin I, *BNP* brain natriuretic peptide (BNP), *MDA* cardiac tissue malondialdehyde, *TAC* cardiac tissue total antioxidant capacity, *TNF-α* cardiac tissue tumor necrosis factor alpha; (TNF-α), *IL-1β* cardiac tissue interleukin 1-beta; and casp-3, cardiac tissue caspase-3. Pearson r is Pearson correlation coefficient. Positive correlation indicate parallel relationship and negative correlation indicates inverse relationship

## Discussion

Low molecular weight heparins including ENX are frequently used to mitigate the risk of venous thromboembolism in cancer patients. In addition to that, LMWHs have been clinically shown to provide a survival benefit that is attributed to not only its anti-thrombotic activity but also to anti-neoplastic effects [28, 29]. In this study, we were looking for another potential role of ENX in attenuating cardiac toxicity of the chemotherapeutic agent, DOX. Our data showed that ENX treatment was able to ameliorate the injury of cardiomyocyte induced by DOX, reduce tissue inflammation and partially restore antioxidant capacity, suggesting significant protective effects for ENX against DOX-induced cardiac tissue damage. Our study, therefore, highlights potential therapeutic advantages for ENX and perhaps LMWHs when treatment is combined with DOX. Clinically, data described herein may promote more effective dose titration in cancer patients treated with DOX when adequate cardioprotection is delivered through ENX therapy.

There has been mixed reports of decreased [31] and increased heart weight [30] following a 2-week DOX course, which were associated with parallel change in heart weight-to-body weight ratio. In our study, DOX chemotherapy contributed a decrease in heart weight, which is perhaps driven by local tissue necrosis as seen in this study, was prevented by ENX treatment, suggesting that ENX can, at least partly, halt tissue gross anatomical changes driven by cardiotoxic effects of DOX. Interestingly, when heart weight was expressed relative to body weight this trend became no longer evident. These effects may have been due to the reduction in heart weight and the concomitant minor drops in body weights in the DOX group, which maintained the heart weight-to-body weight ratio at a constant level.

Cardiac cellular injury in DOX-administered rats was markedly reduced with ENX treatment as indicated by reductions in cTnI release. cTnI, whose release from cardiac myocytes is proportional to the size and extent of cardiac tissue injury [32], has been considered as one of the most reliable and commonly used biomarker for evaluating anthracycline-induced cardiotoxicity [33, 34]. Indeed, DOX administration in the present study induced cardiomyocyte damage which was evidenced by a significant increase in serum level of cTn I relative to controls. Importantly, cTnI, when measured as short as 2 weeks after DOX, appears to be a better and more sensitive marker than BNP in reflecting DOX-induced cardiac injury as changes in serum level of BNP were not significant at the study endpoint. This finding is consistent with results described by Atas et al., 2015 who also showed that the increase in serum level of BNP was not significant when quantified two weeks after DOX injection [35]. One likely explanation underlying this finding is that BNP is considered as a regulatory peptide and is only released in response to ventricular wall distension and cardiac remodeling after injury. These cardiac adaptive mechanisms take time to develop and therefore a two-week timeframe may have been relatively short for these changes to take place. In support of this view are the findings of marked increases in BNP levels which were observed after 6–12 week of DOX administration in rats [36, 37]. The cardioprotective effect of heparin against DOX-induced cardiotoxicity has been previously described in the literature. Nanba et al. (2015) demonstrated that treatment of DOX-administered rats with regular heparin lowered cardiac enzyme and troponin-T (TnT) in effluate taken from rats’ isolated hearts [14]. As far as LMWHs are concerned, a previous animal study has also demonstrated a cardioprotective effect for certoparin sodium against cardiotoxic action of DOX, which was contributed to by reductions in cardiac enzyme activities including creatinine phosphokinase and lactate dehydrogenase in both serum and heart tissues [38]. However, this is the first study to evaluate the cardioprotective effect of ENX in DOX-induced cardiotoxicity, which perhaps indicates that most, if not all, LMWHs may have similar therapeutic benefits when they are used in DOX-treated cancer patients. Future experimental and clinical studies can perhaps compare therapeutic efficacy of a range of LMWHs to determine which therapy provides the greatest cardioprotection against the cardiotoxic effects of DOX.

Consistent with previous studies [39], our data showed that DOX administration in rats caused a significant increase in lipid peroxidation, which was manifested by pronounced elevations in MDA and was also accompanied by a notable decrease in TAC of rats’ cardiac tissue. These effects were not unexpected since previous studies had demonstrated DOX-induced depletion of antioxidant molecules including glutathione (GSH) and exhaustion of cardiac antioxidant enzymes including catalase (CAT), superoxide dismutase (SOD) and glutathione peroxidase (GSH-P) due to excessive consumption by DOX-generated free radicals [40, 41]. The antioxidant activity of ENX was demonstrated in previous studies [42, 43] and was also shown with the LMWH certoparin, which was found to attenuate DOX-mediated cardiac and hepatic lipid peroxidation in rat via lowering MDA, restoring normal levels of the antioxidant molecules GSH and vitamins C and E as well as increasing the activity of antioxidant enzymes SOD, CAT and glutathione peroxidases (GPx) [20]. In our study, although ENX treatment did not significantly alter MDA and TAC as compared to the DOX group, levels were influenced adequately enough to relatively match those observed in the control group, suggesting partial restoration of tissue antioxidant capacity in ENX-treated rats. Higher doses or longer treatment duration may reveal a more pronounced antioxidant potential for ENX that may perhaps extend to upregulating other antioxidant molecules (e.g., GSH, CAT, SOD and/or GSH-P); however, more studies are warranted to confirm this speculation. It will also be of interest to determine the possible mechanisms by which ENX elicits its antioxidant activity in this rat model of DOX-induced cardiotoxicity. However current evidence suggests that the antioxidant effect of heparin and its low molecular weight derivative might be attributed to its structure, being a glycosaminoglycan which acts as a free radical sink [44] or a polyanion with the ability to (1) chelate inorganic ions such as free iron and inhibit free radical generation [45]; or (2) confine SOD to cell surfaces via heparin-binding domain, thus inhibiting tissue injury by free radicals [46].

Cardiac tissue inflammation is a major adverse reaction following DOX exposure that has consistently been shown in animals administered with DOX [47–49]. There is also a substantial evidence which has demonstrated that DOX elicits series of inflammatory responses within the myocardium by upregulating NF-κB and inducing subsequent release of several pro-inflammatory cytokines including TNF-α [11, 50]. Most recently, it has been shown that the progressive increase in pro-inflammatory cytokines within cardiac tissue could be the pathological basis for DOX-induced cardiomyopathy [51]. Consistent with those reports, results of the present study supported a key role for inflammation in the pathogenesis of DOX-induced cardiotoxicity, demonstrating significant elevations in cardiac TNF-α and IL-1β in DOX group as compared to controls. The principal underlying mechanism promoting this increase in inflammatory markers is not fully understood; however, it is possible that impaired tissue antioxidant capacity, heightened levels of ROS and subsequent lipid peroxidation are triggering factors for these changes. Indeed, it has recently been reported that increases in inflammatory mediators were correlated with increased oxidative stress, which is thought to trigger inflammatory reactions via activation of the NF-κB pathway leading to the transactivation of cytokines [49].

Beyond their anticoagulant effects, heparin and ENX have frequently been shown to possess various anti-inflammatory and immunomodulatory properties [17, 18]. The present study demonstrated that treatment with ENX caused significant reductions in cardiac TNF-α and IL-1β levels, suggesting a reliable cytoprotective action for ENX against DOX-mediated release of inflammatory mediators within cardiac tissue. In a rat model of DOX-induced cardiotoxicity, certoparin was also shown to promote a similar reduction in TNF-α [38], which indicates that the observed anti-inflammatory effect may not be limited to ENX. The mechanism by which ENX lowers TNF-α and IL-1β levels following DOX exposure has not been described in this study but current evidence suggests that the anti-inflammatory effect of LMWHs could be due to inactivation of NF-κB pathway, which therefore suppresses subsequent cytokine release [52].

Apoptosis is involved in the pathogenesis of DOX-induced cardiotoxicity [4]. Increased oxidative stress evoked by DOX triggers many signaling pathways including the activation of caspase-3 leading to cardiomyocyte apoptosis [8, 53]. Results from this study, like those reported by Chen et al.*,* 2015, showed significant increases in caspase-3 activity in cardiac tissue following DOX administration [41], an effect which is thought to be driven by a mitochondrial pathway [54]. Interestingly, ENX was able to markedly lower cardiac tissue levels of caspase-3 in DOX-treated rats, suggesting a considerable potential for ENX therapy to modulate cellular apoptosis and therefore alter the course of DOX-mediated cardiac cell death. The exact mechanism by which ENX contributes reductions in caspase-3 in DOX-induced cardiotoxicity has not been determined; however, the anti-apoptotic effect of heparins has been clearly demonstrated in previous studies and been attributed to reductions in the expression of pro-apoptotic proteins such as caspase-3, B-cell lymphoma 2-associated X protein (Bax), apoptosis stimulating fragment (Fas) and stimulation of the anti-apoptotic protein B-cell lymphoma 2 (Bcl-2) [55, 56]. These effects are thought to be promoted by various cellular actions including free radical scavenging activity and/or suppression of the inflammatory response [56]. Taken together, it is therefore possible that our observation of ENX-mediated decreases in the inflammatory mediators, TNF-α and IL-1β, and the partial improvement in the antioxidant capacity of cardiac tissue may have underlined reductions in DOX-mediated increases in cellular caspase-3 level.

Two weeks of DOX administration caused severe histopathological lesions, which were characterized by myocardial swelling, cytoplasmic vacuolization, perinuclear vacuolization, disarray of myocardial fibers and myofibrillar loss. These structural and cellular changes are consistent with several previous studies on DOX-induced cardiomyopathy in rat [57, 58]. The histopathological finding of the present study revealed an ability for ENX to attenuate cardiac tissue lesions driven by DOX as indicated by the marked improvement in CMY severity score compared to the DOX group. A similar cardioprotective effect for the LMWH certoparin has been previously reported [38], where cardiac histopathological changes induced by DOX were almost reverted back to normal myocardial architecture. This, however, was not observed in our study and scores were still higher relative to the control group. It, therefore, remains to be determined if more aggressive treatment with ENX would offer greater cardioprotection on both cellular and structural levels.

Multiple mechanistic pathways are likely to be involved in DOX-induced cardiotoxicity including (1) increased generation of ROS, lipid peroxidation and subsequent oxidative damage to mitochondria, myocytes cell membrane and cellular macromolecules [4, 9]; (2) increased inflammatory responses within the myocardium and heightened release of pro-inflammatory mediators including TNF-α and IL-1β and (3) apoptosis of cardiomyocyte due to mitochondrial dysfunction and cellular degeneration [4, 49]. Data from the current study, therefore, supports a likely role for oxidative stress, inflammation and apoptosis in the pathogenesis of cardiac damage evoked by DOX as these pathways were not only favorably influenced by ENX treatment but were also significantly correlated with the CMY scores measured.

## Conclusion

Enoxaparin treatment effectively attenuates DOX-induced cardiotoxicity via suppression of oxidative stress, inflammation and apoptosis.

## Abbreviations

1β: Interleukin-1 beta
BNP: Brain natriuretic peptide
CMY: Cardiomyopathy
cTNI: cardiac troponin I
DOX: Doxorubicin
ENX: Enoxaparin IL
LMWH: Low molecular weight heparin
MDA: Malondialdehyde NF
NLRP3: NO)-like receptor protein 3
NOD: Nucleotide-binding oligomerization domain
TCA: Total antioxidant capacity
TNF-α: Tumor necrosis factor alpha
κB: Nuclear factor kappa B cell
